# Reconstruction of karyotypic evolution in Saccharum spontaneum species by comparative oligo-FISH mapping

**DOI:** 10.1186/s12870-022-04008-7

**Published:** 2022-12-20

**Authors:** Zhuang Meng, Fei Wang, Quanliang Xie, Rong Li, Haitao Shen, Hongbin Li

**Affiliations:** Key Laboratory of Xinjiang Phytomedicine Resource and Utilization of Ministry of Education, Key Laboratory of Oasis Town and Mountain-basin System Ecology of Xinjiang Production and Construction Corps, College of Life Sciences, Shihezi University, Shihezi, 832003 China

**Keywords:** Chromosome identification, Barcode oligo-FISH, Karyotype evolution, *Saccharum spontaneum*, Chromosomal rearrangement, Genetic relationships

## Abstract

**Background:**

Karyotype dynamics driven by chromosomal rearrangements has long been considered as a fundamental question in the evolutionary genetics. *Saccharum spontaneum*, the most primitive and complex species in the genus *Saccharum*, has reportedly undergone at least two major chromosomal rearrangements, however, its karyotypic evolution remains unclear.

**Results:**

In this study, four representative accessions, i.e., hypothetical diploid sugarcane ancestor (sorghum, x = 10), *Sa. spontaneum* Np-X (x = 10, tetraploid), 2012–46 (x = 9, hexaploid) and AP85–441 (x = 8, tetraploid), were selected for karyotype evolution studies. A set of oligonucleotide (oligo)-based barcode probes was developed based on the sorghum genome, which allowed universal identification of all chromosomes from sorghum and *Sa. spontaneum*. By comparative FISH assays, we reconstructed the karyotype evolutionary history and discovered that although chromosomal rearrangements resulted in greater variation in relative lengths of some chromosomes, all chromosomes maintained a conserved metacentric structure. Additionally, we found that the barcode oligo probe was not applicable for chromosome identification in both *Sa. robustum* and *Sa. officinarum* species, suggesting that sorghum is more distantly related to *Sa. robustum* and *Sa. officinarum* compared with *Sa. spontaneum* species.

**Conclusions:**

Our study demonstrated that the barcode oligo-FISH is an efficient tool for chromosome identification and karyotyping research, and expanded our understanding of the karyotypic and chromosomal evolution in the genus *Saccharum*.

**Supplementary Information:**

The online version contains supplementary material available at 10.1186/s12870-022-04008-7.

## Background

The karyotype is defined as the most general description of the number, morphology and size of all chromosomes in the nucleus, representing the basic genetic information of a eukaryotic species [1, 2]. Comparative karyotypes of related species can be used to establish taxonomic relationships and reveal evolutionary origins [3, 4]. Establishing the karyotype of a eukaryotic species mainly relies on the identification of individual chromosomes. However, the reliable identification of chromosomes has long been as a huge challenge in most non-model species, especially those with large and complex genomes. Up to now, although many plant karyotypes have been established, such karyotypes cannot be used for evolutionary origin studies among related species due to the absence of reliable individual chromosome identification markers [1].

Various cytological techniques have been developed for chromosome identification and karyotype analysis. Among them, chromosome banding and fluorescence in situ hybridization (FISH) were two landmark techniques in the history of cytogenetics. In 1970s, chromosome banding (G-banding) was commonly used to identifying mammalian chromosomes [5], but was not available in most plants [6, 7]. In 1980s, FISH emerged and immediately became the most important technique for chromosome identification in animal and plant species [8–11]. Repeated sequences and bacterial artificial chromosome (BAC) clones are the two most popular FISH probes for chromosome identification and karyotype analysis [12–16]. However, these two probe types are time consuming to develop and are not used as a universal chromosome identification marker among related species or different genotypes [17]. Recently, a new generation FISH probe type based on synthetic oligonucleotide (oligo) has been developed [18, 19], and successfully used in chromosome identification and karyotype analysis of plant species [1, 2, 20–26]. Oligo-based chromosome painting and barcode are the two major probe classes for chromosome identification in plants. The difference between two types of probes is that the FISH signal generated by the former can cover an entire chromosome [27], but each probe allows the identification of only one chromosome and is more expensive, while the latter can simultaneously distinguish all chromosomes in the metaphase cells by one FISH experiment and low cost [4], but only provides limited information on chromosome variations.

Sugarcane is one of the most important economic crops with an annual value of US$90 billion and provides 80% of the world’s sugar and 40% of ethanol [28]. Published genomic studies show that sugarcane and sorghum genomes are mostly collinear in the genic regions, sharing a common ancestor about 8–9 million years ago [29–31]. To date, no natural diploid sugarcane has been found, and sorghum (2n = 2x = 20) can be tentatively regarded as the hypothetical diploid sugarcane ancestor type. *Saccharum spontaneum* is a founding *Saccharum* species with wide variation in chromosome numbers (2n = 4x = 40 to 2n = 8x = 128) [28]. Previous studies have demonstrated that *Saccharum spontaneum* species exhibits three basic chromosome numbers, x = 8, x = 9 or x = 10 [28, 29, 32–34]. Although both *Saccharum spontaneum* (x = 8) and (x = 10) genomes have been sequenced [28, 29], the karyotypic and chromosomal evolution in *Saccharum spontaneum* species is still unclear at the cytogenetic level. In this study, four representative accessions were selected for karyotype evolution studies, i.e., hypothetical diploid sugarcane ancestor (*Sorghum bicolor* BTx623, x = 10), *Sa. spontaneum* Np-X (x = 10, tetraploid), *Sa. spontaneum* 2012–46 (x = 9, hexaploid) and *Sa. spontaneum* AP85–441 (x = 8, tetraploid). We report the development of oligo-based barcode FISH probe based on the *So. bicolor* genome that can be used to simultaneously distinguish all chromosomes of both *So. bicolor* BTx623 and *Sa. spontaneum* Np-X in one cell. Using this barcode oligo probes, we revealed two chromosomal rearrangement events leading to the reduction of basic chromosome number in *Sa. spontaneum* species from 10 to 9 to 8. Accurate karyotypes based on individually identified chromosomes were established in these four accessions, and the results show that although chromosomal rearrangements resulted in greater variation in relative lengths of some chromosomes, all chromosomes maintained a conserved metacentric structure from hypothetical diploid sugarcane ancestor (sorghum, x = 10) to *Sa. spontaneum* AP85–441 (x = 8). Our results demonstrated that oligo-based barcode FISH is a powerful tool for studying karyotype evolution in sugarcane.

## Results

### Development of barcode oligo-FISH probes for chromosome identification

**Fig. 1:**
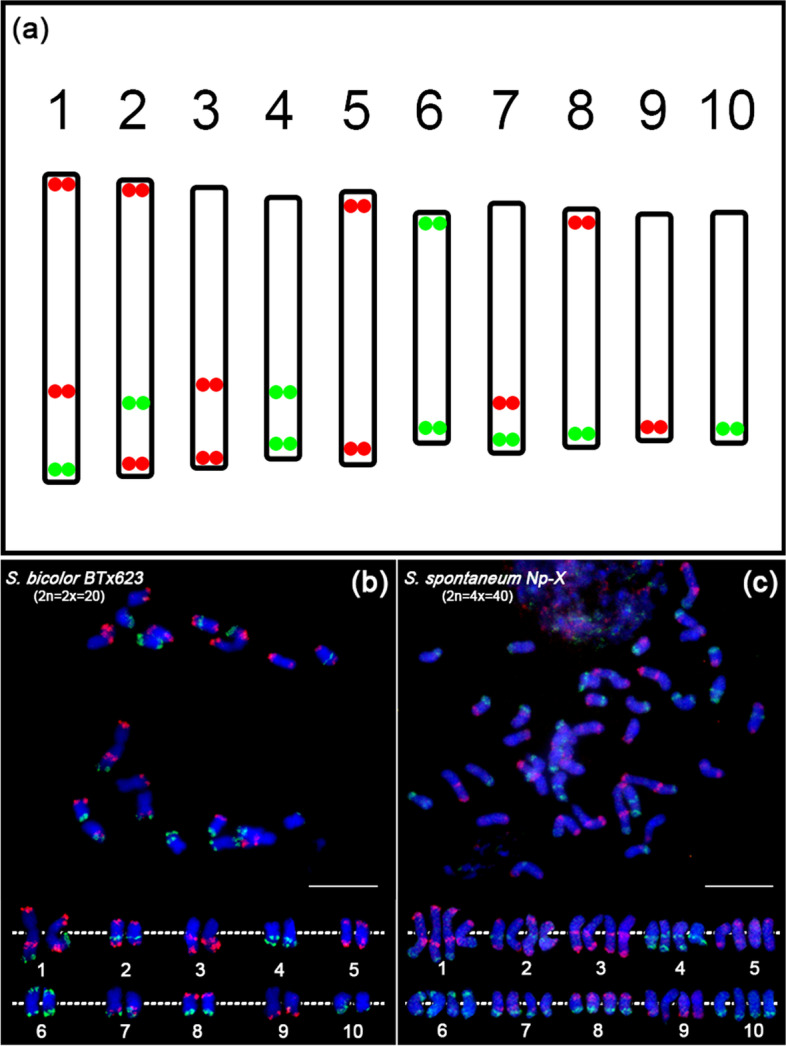
Development of barcode oligo-FISH probes for *So. bicolor* and *Sa. spontaneum* chromosome identification. **a** Oligos were selected from a total of 20 chromosomal regions on 10 sorghum chromosomes (11 red regions and 9 green regions). All 10 basic chromosomes can be distinguished simultaneously based on number and location of the red/green signals. The red/green dots represent the color of the signals produced by the probe, and its position on the chromosome is drawn based on the data in Table S1. **b** FISH analysis of diploid *So. bicolor* BTx623 (2n = 2x = 20) using the barcode oligo probes on mitotic metaphase chromosomes. **c** FISH analysis of autotetraploid *Sa. spontaneum* Np-X (2n = 4x = 40) using the barcode oligo probes on mitotic metaphase chromosomes. The top panels show a complete metaphase cell from *So. bicolor* and *Sa. spontaneum*, respectively. The bottom panel shows the homologous chromosomes of each of the 10 chromosomes digitally excised from the same cell. The centromeres positions of the chromosomes are aligned by a white dotted line. Bars = 10 μm

To distinguish each of the ten basic chromosomes of both sugarcane and sorghum, we designed 20 oligo-FISH probes according to the sorghum assembly (https://www.ncbi.nlm.nih.gov/assembly/GCF_000003195.3). These 20 probes contain 31,360 oligonucleotides (59-nt), and are derived from 20 different regions on 10 sorghum chromosomes. The 20 oligo-FISH probes produced 11 red signals and 9 green signals, which can be used as a “barcode” to simultaneously identify all ten basic chromosomes in the same metaphase cells (Fig. 1a). Each probe contains 1568–1960 oligos and spans 2–2.4 Mb of DNA sequence to ensure consistent signal intensity (Table S1). Several chromosomal arms (chromosome 1, 2, 3, 4 and 7) contain two signals, and the distance between the two regions on the same chromosome arm is 6-20 Mb to ensure that the two signals are separated (Table S1).

### Chromosome identification in diploid *so. Bicolor* (x = 10) and autotetraploid *Sa. Spontaneum* (x = 10)

The oligo probes were labeled either directly by fluorescent dyes TAMRA (red) or FAM (green), respectively. Then, those 20 probes were hybridized to the somatic metaphase chromosomes prepared from diploid *So. Bicolor* BTx623 (2n = 2x = 20). The red and green FISH signals derived from the oligo probes matched to the pre-designed patterns in the genome (Fig. 1a and b). The FISH results showed that the signals formed a specific barcode on each chromosome of *So. bicolor*, which could simultaneously distinguish all 10 basic chromosomes in a cell. We then conducted oligo-FISH in autotetraploid *Sa. spontaneum* Np-X (2n = 4x = 40). FISH assays using these *So. bicolor* probes in Np-X demonstrated a clear signal (Fig. 1c), validating the feasibility of this probe set for application in *Sa. spontaneum* FISH experiment. We observed four identical copies of each of the 10 chromosomes from Np-X. When checking the signal distributions, we found similar localizations for each probe in *Sa. spontaneum* Np-X and that in *So. bicolor* BTx623 (Fig. 1b and c), confirming that *Sa. spontaneum* (x = 10) has a similar karyotype to that of *So. Bicolor (x = 10)*.

### Chromosome identification in *Sa. Spontaneum* 2012–46 (x = 9) and AP85–441 (x = 8)

**Fig. 2:**
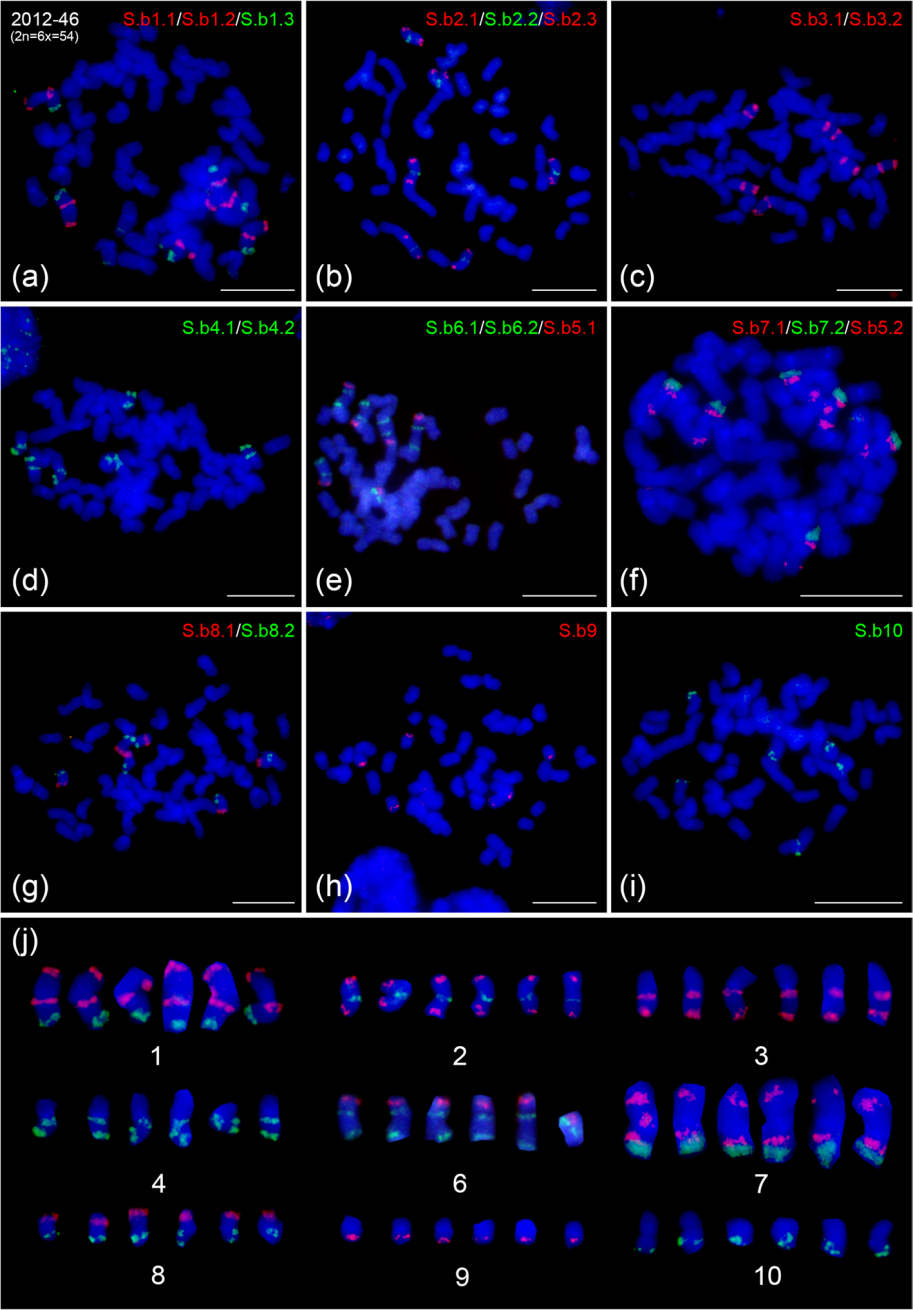
Fluorescence in situ hybridization (FISH) analysis in *Sa. spontaneum* 2012–46 (x = 9). **a-d, g-i** FISH mapping of chromosome 1, 2, 3, 4, 8, 9 and 10 specific barcode oligo probes on a mitotic metaphase cell of hexaploid 2012–46 (2n = 6x = 54), respectively. **e** FISH assay of chromosome 6 specific barcode oligo probes and S.b5.1 probe in hexaploid 2012–46. **f** FISH assay in hexaploid 2012–46 using chromosome 7 specific barcode oligo probes and S.b5.2 probe. **j** shows the 6 homologous chromosomes of each of the 9 *Sa. spontaneum* 2012–46 chromosomes digitally excised from a-i. Bars = 10 μm

To reveal the karyotype evolution of basic chromosome reduction from x = 10 to x = 8 in sugarcane, we conducted comparative FISH mapping in *Sa. spontaneum* 2012–46 (2n = 6x = 54) and AP85–441 (2n = 4x = 32) using the barcode oligo probes developed in *So. bicolor*. In 2012–46, we found that chromosome 5 has undergone fission and subsequent translocation to chromosomes 6 and 7, respectively (Fig. 2e and f). The signal distributions on other seven basic chromosome (i.e., chromosome 1, 2, 3, 4, 8, 9 and 10) in *Sa. spontaneum* 2012–46 (Fig. 2a-d and g-i) were identical to those in both *So. bicolor* BTx623 (Fig. 1b) and *Sa. spontaneum* Np-X (Fig. 1c). This fission and translocation event led to a reduction of the basic chromosome number from x = 10 to x = 9 in *Sa. spontaneum* species.

**Fig. 3:**
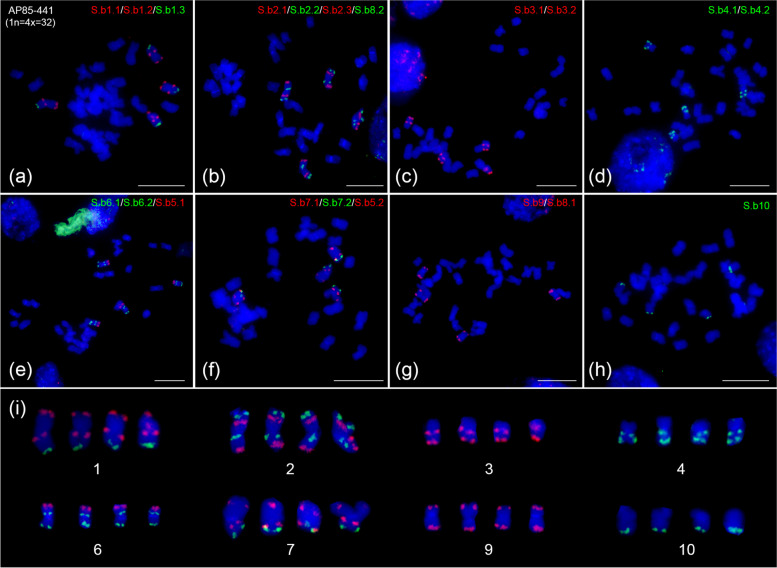
Fluorescence in situ hybridization (FISH) analysis in *Sa. spontaneum* AP85–441 (x = 8).**a, c, d, h** FISH mapping of chromosome 1, 3, 4 and 10 specific barcode oligo probes on a mitotic metaphase cell of tetraploid AP85–441 (1n = 4x = 32), respectively. **b** FISH mapping of chromosome 2 specific barcode oligo probes and S.b8.2 probe in tetraploid AP85–441. **e** FISH mapping of chromosome 6 specific barcode oligo probes and S.b5.1 probe in tetraploid AP85–441. **f** FISH mapping of chromosome 7 specific barcode oligo probes and S.b5.2 probe in tetraploid AP85–441. **g** FISH mapping of chromosome 9 specific barcode oligo probes and S.b8.1 probe in tetraploid AP85–441. **i** shows the 4 homologous chromosomes of each of the 8 *Sa. spontaneum* AP85–441 chromosomes digitally excised from a-h. Bars = 10 μm

In AP85–441, in addition to the fission and translocation event among chromosome 5, 6 and 7 (Fig. 3e and f), similarly, chromosome 8 also split into two segments and subsequently translocated to chromosomes 2 and 9, respectively (Fig. 3b and g). The FISH signals generated on other four basic chromosome (i.e., chromosome 1, 3, 4 and 10) in *Sa. spontaneum* AP85–441(Fig. 3a, c, d and h) were also identical to those in *So. bicolor* BTx623 (Fig. 1b), *Sa. spontaneum* Np-X (Fig. 1c) and 2012–46 (Fig. 2a, c, d and i). The fission and translocation event among chromosome 8, 2 and 9 subsequently led to a further reduction of the basic chromosome number from x = 9 to x = 8 in *Sa. spontaneum* species.

### The karyotypes of *so. Bicolor* and *Sa. Spontaneum* species

**Fig. 4:**
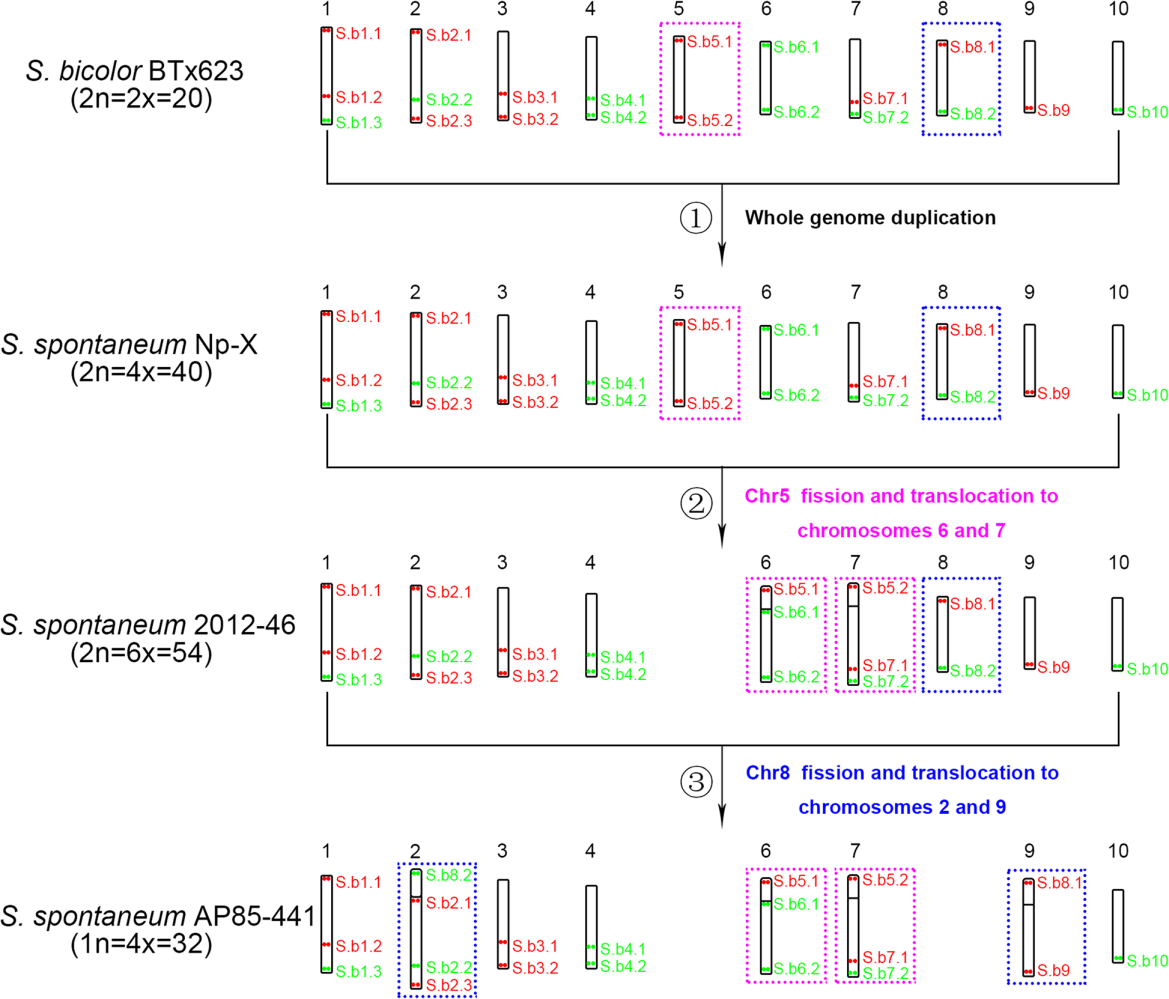
Schematic illustration of karyotype evolution in sugarcane. Ideograms illustrating showing karyotype evolution among putative sugarcane diploid ancestor sorghum BTx623 (2n = 2x = 20), *Sa. spontaneum* Np-X (2n = 4x = 40), 2012–46 (2n = 6x = 54) and AP85–441 (1n = 4x = 32). The position of the oligo probes on the chromosome were drawn based on the FISH results. The pink dotted rectangles represent the chromosomes involved in the fission and translocation event of chromosome 5. The blue dotted rectangles represent the chromosomes involved in the fission and translocation event of chromosome 8

We then developed the karyotypes in *So. bicolor* BTx623, *Sa. spontaneum* Np-X, 2012–46 and AP85–441 based on oligo-FISH chromosome identification results. Each karyotype was developed based on measurements of all chromosomes in 10 complete metaphase cells without apparent chromosomal morphological distortion. The results showed that all the chromosomes are morphologically conserved, and are metacentric in BTx623, Np-X, 2012–46 and AP85–441 (Table 1) (1.02 < arm ratio < 1.34) [35]. According to the karyotype data, we found that although the basic chromosome number in both putative diploid ancestor sorghum BTx623 and autotetraploid sugarcane Np-X is 10, the relative length of chromosomes has a major variation. Except the relative length order (RLO) of the longest chromosomes 1, 2 and 3 did not change, the remaining seven chromosomes were changed. Chromosome 4 is the fourth longest and chromosome 8 is the shortest in BTx623, but in Np-X chromosome 9 is the fourth longest and chromosome 6 is the shortest (Table 1). The comparative karyotype analysis between Np-X (x = 10) and 2012–46 (x = 9) showed that the RLO of chromosomes 1, 7, 10 and 8 were unchanged, and chromosome 6 changed from the shortest to the third longest chromosome due to the fission and translocation event among chromosome5, 6 and 7 (Table 1). Finally, we found that the karyotype changed dramatically as the basic chromosome number decreased again from 2012 to 46 (x = 9) to AP85–441(x = 8). The RLO of all chromosomes were changed except chromosome 6, chromosome 2 changed from the sixth to the longest chromosome due to the fission and translocation event among chromosome8, 2 and 9, and chromosome 10 becomes the shortest. To present an overview of the karyotype evolution feature, an integrated schematic was drawn with the position of the barcode oligo probes (Fig. 4) based on its relative position (Table S1) and FISH results (Figs. 1, 2 and 3).

**Table 1:** Relative lengths and arm ratios of mitotic metaphase chromosomes of *So. bicolor* and three *Sa. spontaneum* accessions

Chr.	*So. Bicolor* BTx623 (2n = 2x = 20)		*Sa. spontaneum* Np-X^a^ (2n = 4x = 40)		*Sa. spontaneum* 2012–46 (2n = 6x = 54)		*Sa. spontaneum* AP85–441 (2n = 4x = 32)	
	Arm ratio	Relative length (%)	Arm ratio^b^	Relative length^c^ (%)	Arm ratio	Relative length (%)	Arm ratio	Relative length (%)
1	1.34 ± 0.24	14.52 ± 1.62	1.08 ± 0.02	13.24 ± 0.09	1.13 ± 0.10	14.61 ± 1.09	1.15 ± 0.15	15.11 ± 2.15
2	1.22 ± 0.17	10.91 ± 1.10	1.11 ± 0.01	10.65 ± 0.06	1.18 ± 0.19	10.12 ± 1.20	1.12 ± 0.10	17.68 ± 2.29
3	1.12 ± 0.09	10.98 ± 0.65	1.12 ± 0.07	10.60 ± 0.60	1.18 ± 0.12	14.29 ± 1.36	1.12 ± 0.10	10.85 ± 0.54
4	1.19 ± 0.11	9.64 ± 0.59	1.12 ± 0.02	9.62 ± 0.11	1.23 ± 0.20	12.12 ± 0.98	1.21 ± 0.18	10.24 ± 0.73
5	1.16 ± 0.14	9.60 ± 0.49	1.02 ± 0.01	9.18 ± 0.09	/	/	/	/
6	1.28 ± 0.25	9.11 ± 0.54	1.28 ± 0.10	8.54 ± 0.33	1.15 ± 0.13	13.10 ± 0.88	1.13 ± 0.11	13.16 ± 0.88
7	1.14 ± 0.11	9.48 ± 0.64	1.07 ± 0.03	9.84 ± 0.20	1.22 ± 0.16	11.40 ± 0.77	1.25 ± 0.17	12.27 ± 1.23
8	1.16 ± 0.14	8.49 ± 0.68	1.06 ± 0.01	8.82 ± 0.05	1.14 ± 0.10	7.33 ± 0.77	/	/
9	1.15 ± 0.12	8.58 ± 0.79	1.12 ± 0.02	10.19 ± 0.10	1.15 ± 0.12	7.76 ± 0.97	1.26 ± 0.21	11.32 ± 0.61
10	1.14 ± 0.06	8.70 ± 0.81	1.19 ± 0.05	9.33 ± 0.32	1.13 ± 0.09	9.27 ± 1.16	1.12 ± 0.10	9.38 ± 0.66

^a^The Np-X karyotype data were obtained from our previous published article (Meng et al. 2019, Theoretical and Applied Genetics)

^b^Arm ratio, length of the long arm/length of the short arm

^c^Relative length, chromosome length/genome length

Measurement was performed on each chromosome in 10 metaphase cells

### Application of the barcode oligo probes in *Sa. Robustum and Sa. Officinarum* species

**Fig. 5:**
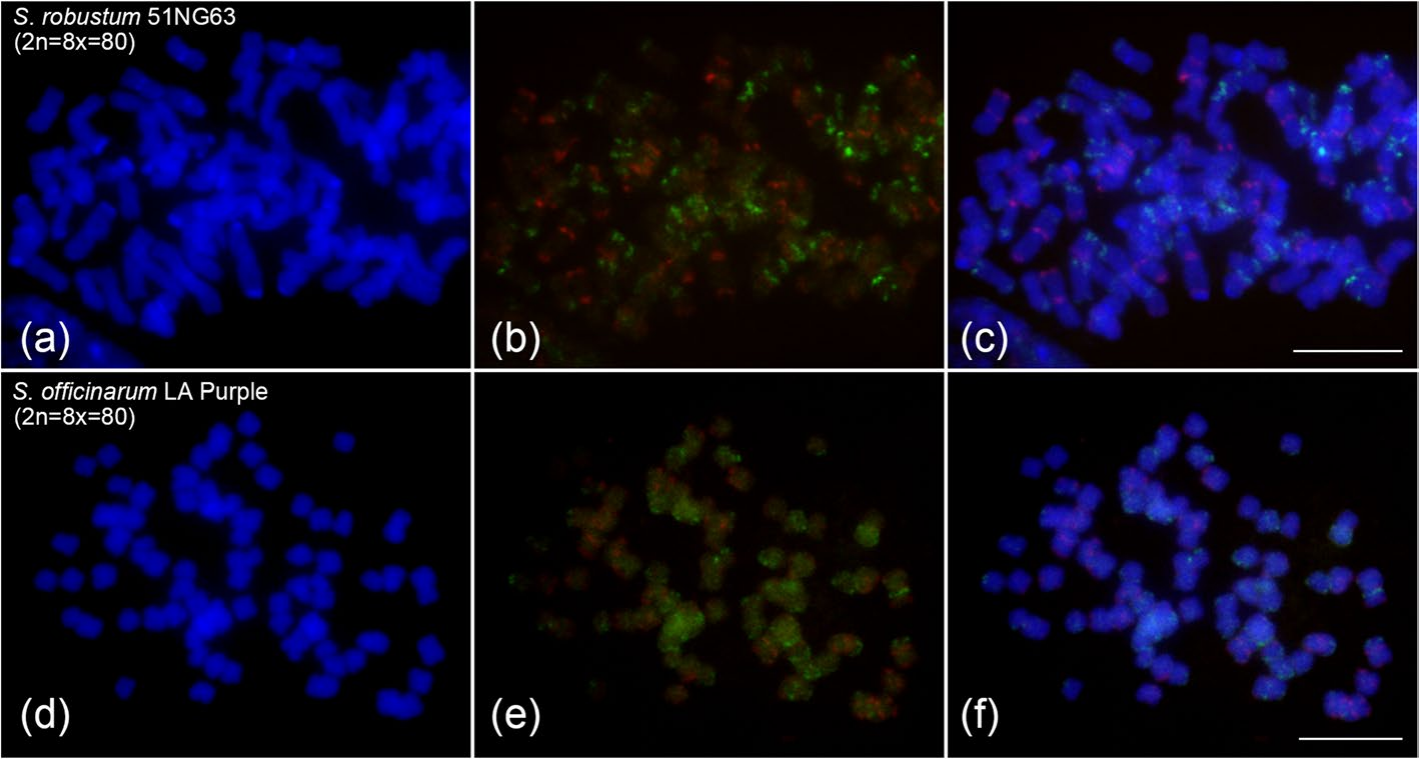
FISH mapping of the brocade oligo probes on metaphase chromosomes of *Sa. robustum* and *Sa. officinarum* (x = 10). **a-c** FISH mapping of *Sa. robustum* 51NG63 (2n = 8x = 80). **d-f** FISH mapping of *Sa. officinarum* LA Purple (2n = 8x = 80). Images in first column: chromosomes counterstained with DAPI; Images in the second column: digitally separated FISH signals derived from the barcode oligo probes; Images in the third column: complete metaphase cells hybridized with the barcode oligo probes. Bars = 10 μm

We were intrigued by the applicable potential of the barcode probes for chromosome identification in *Sa. robustum* (x = 10) *and Sa. officinarum* (x = 10). We first examined the barcode oligo probes in hybridization to metaphase chromosomes prepared from *Sa. robustum* 51NG63 (2n = 8x = 80). The FISH result showed that the barcode probes only generated consistent and distinct signal patterns on some chromosomes (Fig. 5a-c), but most of the FISH signals were not as strong as those on *So. bicolor* (Fig. 1b) and *Sa. spontaneum* chromosomes (Figs. 1c, 2 and 3). In addition, we observed background noise signals on most of chromosomes, and even no unambiguous signals on several chromosomes. Subsequently, we performed FISH assay using the barcode oligo probes in *Sa. officinarum* LA Purple (2n = 8x = 80). The barcode oligo-FISH probes produced punctuated signals and stronger background noise signals on almost all chromosomes (Fig. 5d and f), which cannot be used to identify chromosomes in LA Purple. Therefore, the *So. bicolor* probes developed in this study were not be used for chromosome identification in both *Sa. robustum* and *Sa. officinarum* species.

## Discussion

*Saccharum spontaneum* is the most primitive and complex species in the genus *Saccharum* with highest level of genetic diversity (nearly 40 chromosome-number types, 2n = 40–128). Great efforts have been made in chromosome identification [34, 36–38], karyotyping research [21, 39] and genomics studies [28, 29, 40, 41] of *Sa. spontaneum* species because of its importance in sugarcane breeding. Our previous studies have demonstrated the existence of three basic chromosome numbers of 8, 9 and 10 in *Sa. spontaneum* species [32, 33], and established karyotypes with basic chromosome numbers of 8 [39] and 10 [32], respectively. However, these karyotypes were not based on a universal chromosome identification system and cannot be used for comparative karyotype studies. Comparative karyotype analysis can provide key cytogenetic information on the phylogenetic relationships and evolutionary origins [42, 43], helping us reconstruct the evolutionary history that shaped karyotype diversity in *Sa. spontaneum* species. Using a unified barcode oligo-FISH system, we conducted comparative karyotype analysis and reconstructed a panorama of karyotype evolution from hypothetical diploid sugarcane ancestor (sorghum) to extant *Sa. spontaneum* species (Fig. 4, Table 1). As we expected, the relative length of most chromosomes varied dramatically because of two chromosome fission and translocation events (Table 1). We also detected unexpected all chromosomes maintain a conserved arm ratio structure (metacentric, 1.02–1.34) during karyotype evolution. The two published *Sa. spontaneum* (x = 8 and x = 10) genomes also show that almost all chromosomes were metacentric or submetacentric, which was consistent with our cytology results [28, 29]. According to normal logic, all chromosomes were metacentric type in *Sa. spontaneum* (x = 10). After two fission and fusion of Chr5 and Chr8, the arm ratio on the related chromosomes should be changed in *Sa. spontaneum* (x = 9 and x = 8), but our cytology results showed that no major variation occurred. We speculate that there may be two reasons: 1: chromosomes 5 and 8 are smaller so that chromosomal rearrangements are not sufficient to change the arm ratio of the related chromosomes; 2. the related chromosomes involved in chromosomal rearrangements undergo evolutionary centromeric repositioning. Most recently, Yu et al., (2021) developed the karyotype in four species, including *Saccharum. officinarum*, *Saccharum. robustum*, *Narenga. porphyrocoma*, *Erianthus. rockii* and *Erianthus. fulvus* using chromosome painting, and found that although the *Saccharum* complex undergoes several chromosome rearrangements but all chromosomes were also metacentric or submetacentric [21]. Combined with our study, we thought it will be interesting to investigate possible mechanisms for the evolution of centromeres and karyotypes in the complex genus *Saccharum* and related genera.

An important use for the oligo-based barcode markers is for investigating genetic relationship between different species within a genus or relative genera. The specificity and intensity of the signal generated by barcode oligo probes were dissimilar between different species or genera. In general, oligo probes produce relatively consistent signals in closely related species, whereas in more distant species they produce dispersive or less specific signals or even no signal. The barcode oligo probes derived from *Oryza. sativa* has been used for studying species evolution. According to the specificity and intensity of the signal generated by the barcode probe, they revealed that the AA, BB and CC *Oryza* varieties were closely related to *O. sativa* than EE *Oryza* variety, and FF *Oryza* variety was the farthest [4]. Similarly, Braz et al., (2020) also confirmed that maize barcode oligo probes can generate consistent and specific signals in several closely related *Zea. mays* subspecies and wild *Zea* species (divergence time ~ 0.15 MYA) while producing strong background noise signals in more distantly related *Tripsacum dactyloides* (divergence time ~ 4.5 MYA) and *Sorghum bicolor* (divergence time ~ 12 MYA) [44]. In the present study, our cytology results showed that sorghum (x = 10) barcode oligo probes produced more specific signals in *Sa. spontaneum* species with different basic chromosome number (x = 10, 9 and 8), more dispersive or less specific signals in *Sa. robustum* (x = 10), and strong background noise signals in *Sa. officinarum* species (x = 10), suggesting that sorghum may be more closely related to *Sa. spontaneum* species than to *Sa. robustum* species, and most distantly related to *Sa. officinarum* species. The previously published *Sa. spontaneum* (x = 8) genome study showed that *Sa. spontaneum* and *So. bicolor* genomes are mostly collinear in the genic regions (~ 90%), which suggested that *So. bicolor* was closely related to *Sa. spontaneum* species [29]. Moreover, the recently published *Sa. spontaneum* (x = 10) genome study showed that *Sa. spontaneum* split from *Sa. officinarum* about 1.6 Mya [28], while the *Sa. officinarum* has been considered to be domesticated from *Sa. robustum* [45]. This implies that *So. bicolor* was more distantly related to *Sa. robustum* and *Sa. officinarum* than *Sa. spontaneum*. These findings are consistent with our evolutionary hypothesis. In general, genetic markers were reliable and common method to investigated phylogenetic relationships. Compared with genetic markers, the barcode oligo-FISH tool has the advantage of being able to clearly and intuitively visualize the evolutionary differences of each chromosome or chromosome segment between different species, thus playing a role in investigating the phylogenetic relationship. In the future, a combination of genetic markers and barcode oligo-FISH tools can be used to more precisely infer phylogenetic relationships between different species. This study also reminds us that if we want to develop a set of oligo-based markers that can be used to identify the chromosomes of distantly related species or genera in the future, we must select the conserved regions shared by the genomes of related species to design as many oligos as possible for the purpose of chromosome identification. Together, the cytological analysis by barcode oligo-FISH provides us a reliable experimental tool for the study of phylogenetic relationship among different species or genera. Further applications of this technology will provide deeper insights into evolutionary origins among plant species/genera with large and complex genomes.

## Conclusions

In this study, we developed a set of barcode oligo probes that can be used to accurately distinguish all chromosomes in both sorghum and *Sa. spontaneum* species. Using this barcode oligo probes, we reconstructed the karyotype evolutionary history from hypothetical diploid sugarcane ancestor (sorghum, x = 10) to extant *Sa. spontaneum* species (x = 10, 9 and 8). The karyotype date show that although chromosomal rearrangements resulted in large changes in relative lengths of some chromosomes, all chromosomes maintained a conserved metacentric structure during karyotype evolution. Moreover, we also found that sorghum (x = 10) is more distantly related to *Sa. robustum* and *Sa. officinarum* (x = 10) species compared with *Sa. spontaneum* (x = 10, 9 and 8) species by barcode oligo-FISH. Our study confirms that barcode oligo-FISH is a powerful tool for sugarcane chromosomal research, and expanded our understanding of the karyotypic evolution in complex *Sa. spontaneum* species.

## Methods

### Plant materials

The materials used in this study included *So. bicolor* BTx623 (2n = 2x = 20), *Sa. spontaneum* Np-X (2n = 4x = 40), *Sa. spontaneum* 2012–46 (2n = 6x = 54), *Sa. spontaneum* AP85–441 (1n = 4x = 32, generated from anther culture of autooctoploid SES208), *Sa. robustum* 51NG63 (2n = 8x = 80) and *Sa. officinarum* LA Purple (2n = 8x = 80). All of the plants were grown in the greenhouse of Fujian Agriculture and Forestry University (Fuzhou, Fujian Province, China) with a 16 h light/8 h dark photoperiod at 30 °C.

### Chromosome preparation

Mitotic chromosome spreads were prepared as previously described [39] with several modifications. Root tips were harvested from sorghum and sugarcane, pretreated with nitrous oxide at a pressure of 10.9 atm (∼160 psi) for 1.5 h, and then fixed in Carnoy’s fixative (3 ethanol:1 acetic acid) at − 20 °C until use. Subsequently, an enzyme mixture (4% cellulase, 1% pectolyase Y23 and 2% pectolyase) was used to digest the root tips for 1 h at 37 °C, and then squashed with a cover slip on glass slide. After that, the glass slides were quickly frozen in liquid nitrogen, and then the cover slips were removed. After naturally dried, the glass slides of chromosome spreads were screened with an Olympus CX33 phase-contrast microscope. Finally, glass slides with good mitotic metaphase chromosome spreads were selected for FISH experiments.

### Design and synthesis of the barcode oligo libraries

The barcode oligo libraries were developed using a previously published pipeline [18] with several modifications. Briefly, the repetitive sequences in the sorghum genome were filtered using RepeatMasker and remaining sequences were then divided into oligos (59 nt) with a step size of 5 nt. Then, those oligos was mapped to the sorghum genome and removed oligos with duplicates in the genome (75% similarity over all 59 nt). Oligos with dTm > 10 (dTm = melting temperature Tm − hairpin Tm) were selected to build a probe pool. For the barcode oligo probes, target regions with relatively high oligo density were selected based on the density distribution profile across the chromosomes. Finally, we selected oligos from 20 chromosomal regions to form a specific barcode marker for all 10 sorghum chromosomes (Fig. 1a). The oligo libraries were synthesized by MYcroarray (Ann Arbor, MI, USA).

### Oligo-FISH

FISH procedure was conducted following published protocols with several modifications [32]. Briefly, the oligo probes were labeled with TAMRA-red or FAM-green (direct) according to previously published PCR protocol [46], and then hybridized to metaphase chromosomes. After hybridization, chromosomes were counterstained with 4,6-diamidino-2-phenylindole (DAPI) in VectaShield antifade solution (Vector Laboratories). FISH signal and chromosome images were captured using an Olympus DP80 CCD camera attached to an Olympus BX63 fluorescence microscope. All images were processed with cellSens Dimension 1.9 software. The final contrast of the images was processed and adjusted using Image-Pro Plus and Adobe Photoshop CC software.

### Karyotyping

For karyotyping assay, 10 complete metaphase cells of BTx623, Np-X, 2012–46 and AP85–441 without apparent chromosomal morphological distortion were analyzed using Image-Pro Plus software version 6.0. We measured the sizes of short (S) and long (L) arms of individual chromosomes, and then calculated the arm ratio (*AR* = *L*/*S*), total length of each chromosome (*tl* = *S* + *L*), total length of the entire set of chromosomes (*TL* = ∑*tl*), and relative chromosome length (*RL* = *tl*/*TL* × 100). Chromosomal knobs were identified as DAPI-positive bands. Chromosome type were classified based on arm ratio following previously published methods [35].

## Supplementary Information

**Additional file 1:** **Table S1.** Characterizations of barcode oligo probes designed based on the sorghum genome assembly.

## Data Availability

All data in this work are available within the manuscript or are available in additional files.
