# Supplementary material for: Reconstruction of karyotypic evolution in Saccharum spontaneum species by comparative oligo-FISH mapping
Source: BMC Plant Biol. 2022 Dec 20;22:599. doi: 10.1186/s12870-022-04008-7 (PMC9764494; doi:10.1186/s12870-022-04008-7)
Supplement: Supplementary file 1 — Additional file 1: Table S1. Characterizations of barcode oligo probes designed based on the sorghum genome assembly. [file 12870_2022_4008_MOESM1_ESM.docx]

Table S1. Characterizations of barcode oligo probes designed based on the sorghum genome assembly.

| *S. bicolor* chromosome | FISH probe | Start position (Mb) | End position (Mb) | Number of oligos | Region length (Mb) | Density  (oligos/Mb) | Probe color |
| --- | --- | --- | --- | --- | --- | --- | --- |
| 1 | S.b1.1 | 0.0 | 2.0 | 1568 | 2.0 | 784 | Red |
| 1 | S.b1.2 | 55.0 | 57.0 | 1568 | 2.0 | 784 | Red |
| 1 | S.b1.3 | 77.0 | 79.0 | 1568 | 2.0 | 784 | Green |
| 2 | S.b2.1 | 0.0 | 2.0 | 1568 | 2.0 | 784 | Red |
| 2 | S.b2.2 | 56.0 | 58.3 | 1960 | 2.3 | 852 | Green |
| 2 | S.b2.3 | 74.0 | 76.0 | 1568 | 2.0 | 784 | Red |
| 3 | S.b3.1 | 50.0 | 52.0 | 1568 | 2.0 | 784 | Red |
| 3 | S.b3.2 | 72.0 | 74.0 | 1568 | 2.0 | 784 | Red |
| 4 | S.b4.1 | 49.0 | 51.2 | 1568 | 2.2 | 713 | Green |
| 4 | S.b4.2 | 64.5 | 66.7 | 1568 | 2.2 | 713 | Green |
| 5 | S.b5.1 | 2.0 | 4.0 | 1568 | 2.0 | 784 | Red |
| 5 | S.b5.2 | 65.5 | 67.5 | 1568 | 2.0 | 784 | Red |
| 6 | S.b6.1 | 0.0 | 2.1 | 1568 | 2.0 | 784 | Green |
| 6 | S.b6.2 | 57.0 | 59.1 | 1568 | 2.1 | 747 | Green |
| 7 | S.b7.1 | 52.6 | 55.0 | 1568 | 2.4 | 653 | Red |
| 7 | S.b7.2 | 61.0 | 63.4 | 1568 | 2.4 | 653 | Green |
| 8 | S.b8.1 | 2.0 | 4.0 | 1568 | 2.0 | 784 | Red |
| 8 | S.b8.2 | 58.5 | 60.7 | 1568 | 2.2 | 713 | Green |
| 9 | S.b9 | 55.3 | 57.3 | 1568 | 2.0 | 784 | Red |
| 10 | S.b10 | 57.0 | 59.0 | 1568 | 2.0 | 784 | Green |
